# The Interrelation Between Peripersonal Action Space and Interpersonal Social Space: Psychophysiological Evidence and Clinical Implications

**DOI:** 10.3389/fnhum.2021.636124

**Published:** 2021-02-26

**Authors:** Yann Coello, Alice Cartaud

**Affiliations:** Univ. Lille, CNRS, Lille, UMR 9193—SCALab—Sciences Cognitives et Sciences Affectives, Lille, France

**Keywords:** peripersonal space, social interaction, interpersonal distance, emotion, threat, facial expression, clinical population

## Abstract

The peripersonal space is an adaptive and flexible interface between the body and the environment that fulfills a dual-motor function: preparing the body for voluntary object-oriented actions to interact with incentive stimuli and preparing the body for defensive responses when facing potentially harmful stimuli. In this position article, we provide arguments for the sensorimotor rooting of the peripersonal space representation and highlight the variables that contribute to its flexible and adaptive characteristics. We also demonstrate that peripersonal space represents a mediation zone between the body and the environment contributing to not only the control of goal-directed actions but also the organization of social life. The whole of the data presented and discussed led us to the proposal of a new theoretical framework linking the peripersonal action space and the interpersonal social space and we highlight how this theoretical framework can account for social behaviors in populations with socio-emotional deficits.

## The Functional Representation of The Physical Space

Interacting efficiently with the environment requires anticipating at every moment what behavior can be performed depending on the context. Since motor action is the only way for biological organisms to interact with the environment, the cognitive processes flexibly deployed to produce adaptive behavior must take into account not only the characteristics of the external world but also the state of the body and the potentialities offered by acquired motor experiences. As stated by Clark (2001), “conscious visual experience presents the world to the subject in a richly textured way […] especially apt for, and typically utilized in, the control and guidance of fine-tuned, real-world activity” (pp. 496). This implies that the brain retains a functional representation of the visual space, although at an abstract level, which depends on previous events, actions and outcomes while interacting with the environment (Grüsser, 1983; Previc, 1998). The neurologist Brain (1941) reported the primary description of a functional representation of the visual space, dissociating a “grasping distance” and a “walking distance” from the study of brain-injured patients characterized by a selective impairment of information processing in one of the two spaces. The “grasping” space, in which we can directly interact with the objects at hand, was later labeled peripersonal space (PPS hereafter) by Rizzolatti et al. (1981). In line with Brain’s seminal work, Rizzolatti et al. (1981) discovered, from single-unit electrophysiological studies in macaque monkeys, a particular class of neurons that were responding to both somatosensorial and visual stimuli, but only when the latter were located in the PPS. These bimodal neurons, found in the parietal cortex, the premotor cortex, and the putamen, responded as if they were encoding sensory information within an egocentric frame of reference in relation to the motor system. Their responses were specific to tactile stimulation on the face, the neck, the arm, or the hand as well as to visual stimuli located near these body segments (Rizzolatti et al., 1981; Gentilucci et al., 1988; Graziano, 2017). Objects in the vicinity of the body seem thus more relevant by the possible interactions the body can establish with them (Brozzoli et al., 2011). Accordingly, a function of these bimodal neurons seems to link the sight of objects to expected tactile events. The view of a visual object can indeed generate a prediction that anticipates either a movement of the body toward the object or a movement of the object toward the body. The predictive mechanism requires the location of the visual object or event close to the body to be specifically encoded relative to the location of a particular body segment, i.e., in terms of bodily coordinates (or also somatotopic coordinates or skin-based coordinates), despite the fact that they occur in external space, that is, beyond the boundaries of the body (Brozzoli et al., 2011; De Vignemont, 2018). As a consequence, these neurons contribute to multisensory representations of visual objects that are associated with a specific motor repertoire (Graziano and Gross, 1993; Rizzolatti et al., 1997, 1998). Converging data have been then obtained from a variety of studies that investigated multisensory neurons in the frontoparietal regions showing that they specifically respond to stimuli in the PPS (Rizzolatti et al., 1997; Graziano and Cooke, 2006; Cléry et al., 2015). In line with these findings, lesions in the parietal or premotor regions induced inattention to the contralesional space but specifically in the PPS (Rizzolatti et al., 1983). By contrast, lesions in different regions of the parietal cortex or in the prefrontal cortex can produce neglect of objects in the space far (the extrapersonal space, EPS hereafter). Interestingly, it was reported that not only the view of body segments but also their shadows can contribute to facilitating the processing of sensory information relating to the body or the environment (Galfano and Pavani, 2005). Overall, the results showed that body shadows enhance attention towards stimuli located at the vicinity of body segments (Pavani et al., 2014), suggesting that they contribute to the integration of sensory information from the body and the environment, in particular in the PPS (Pavani and Castiello, 2004).

As regards the functional properties of the PPS and motor activity, it was suggested that objects’ coding in the PPS involves not only the integration of multisensory information relating to objects and the body, but also motor information concerning the actions the objects afford (Rizzolatti et al., 1981; Graziano et al., 1999; Makin et al., 2007). Indeed, brain imaging studies showed that objects processing in the PPS activates the multisensory (visual, auditory, olfactory…) brain areas as well as the somatosensory cortex (Matelli et al., 1985; Grafton et al., 1996; Cardellicchio et al., 2011), but also the sensorimotor network including the posterior parietal cortex and the premotor cortex (Chao and Martin, 2000; Chao et al., 2002; Creem-Regehr and Lee, 2005; Kan et al., 2006; Martin, 2007). Early evidence for a contribution of the motor system to objects coding in the PPS was provided by Proverbio (2012). She reported modulation of the brain cortical activity when human participants were observing images of manipulable objects. Time-frequency analysis of EEG signals revealed a desynchronization of the μ rhythm (8–13 Hz cortical oscillation) over the centro-parietal region, linked to the sensorimotor system and similar to that registered when executing or observing a voluntary motor action (Salenius et al., 1997; Babiloni et al., 1999; Cochin et al., 1999; Llanos et al., 2013). More recently, it was found that μ rhythm desynchronization over the centro-parietal region was present when perceiving manipulable objects, but only when the latter was located in the PPS (Kalénine et al., 2016; Wamain et al., 2016). Moreover, μ rhythm desynchronization reduced progressively when moving from reachable to unreachable stimuli (Wamain et al., 2016), suggesting a continuity between the representation of the PPS and that of the EPS (Bufacchi and Iannetti, 2018). Interestingly, it was demonstrated that, in the presence of manipulable objects in PPS, μ rhythm desynchronization over the centro-parietal region was reduced when the objects evoked not a single one, but multiple motor affordances such as those related to the different grasping and using actions characterizing certain manipulable objects (Kalénine et al., 2016). These observations support the idea that the PPS defines a space for action, which is represented based on sensory and motor-related information similar to that involved in the planning of voluntary motor actions (Coello and Iachini, 2016). In agreement with this, Coello et al. (2008) found that transcranial magnetic stimulation (TMS) applied at low frequency to the left motor cortex interferes with perceptual judgments only for stimuli presented in the PPS. Likewise, Cardellicchio et al. (2011) stimulated magnetically the left primary motor cortex and recorded motor-evoked potentials (MEPs) while participants were observing manipulable and non-manipulable objects located either in their PPS or EPS. They found higher MEPs during the observation of manipulable objects when they were located within the PPS. The findings reported so far are thus consistent with the idea that the motor nature of the PPS reflects the adaptive need of anticipating what may happen near the body and prepare to react in time. Objects in the PPS, depending on their valence, would prompt a motor response either in terms of approach when considering attractive stimuli, or in terms of avoidance when considering threatening stimuli, emphasizing the need to anticipate the available motor resources for rapid behavioral responses (Graziano and Cooke, 2006; Coello and Delevoye-Turrell, 2007; Huang et al., 2012; Iachini et al., 2017; Ruggiero et al., 2017). The relationship between the PPS representation and readiness of the body to act indicates therefore that the representation of the PPS should be flexible.

## The Plasticity of Peripersonal Space Representation

Because we adapt constantly our behavior to the environment in which our body is embedded, the representation of the PPS must be flexible and adapt dynamically to the changing sensorimotor context (Coello et al., 2008). Such change of sensorimotor context occurs when, for instance, we use a tool that extends the motor capabilities of our body. Past studies indeed revealed that manipulating a tool changes how we represent near and far visual spaces (Berti and Frassinetti, 2000; Maravita and Iriki, 2004; Cardinali et al., 2012; Canzoneri et al., 2013; Bourgeois et al., 2014), due to the alteration of the representation of the arm in the body schema resulting from tool-use (Cardinali et al., 2009). Indeed, using a long-handle tool with the hand produces an extension of the representation of arm’s length, as if the tool was suddenly like a body segment (Grüsser, 1983). Evidence for an integration of the tool into the somatosensory cortical representation of the arm came from the observation that tool-use modifies the way the brain represents the metric characteristics of the body segment controlling the tool. For example, it was shown that the use of a tool that elongates the physical length of the arm induced kinematic changes affecting subsequent object-oriented motor actions (Cardinali et al., 2009, 2011). In a sense, kinematic parameters of the reaching movement towards a visual object changed as if the object was perceived as being at a closer location after tool-use than before. Also, tool-use modified the perceived morphology of the arm. When neurotypical individuals were asked to localize simultaneous tactile stimuli on their arm after having used a tool, they localized these tactile stimuli as being more distant from each other than before tool-use (Cardinali et al., 2009; Sposito et al., 2012). These findings indicate that tool-use alters the body schema, and more specifically modifies the somatosensory representation of intrinsic properties of body morphology (Cardinali et al., 2009). As a consequence, it was shown that the manipulation of a tool increased the attention paid to the area where the tooltip was active (Holmes et al., 2007). This effect was the result of a displacement of the receptive fields of the somatosensory neurons of the hand towards the functional part of the tool (Iriki et al., 1996). Because some of these neurons were bimodal, they responded both to tactile stimuli on the hand and to visual stimuli that were at a reachable distance with the tool. Using the tool extended outwards along the tool, the neurons’ tactile receptive fields, as well as the visual receptive fields of the same neurons (Iriki and Sakura, 2008). Thus, these visuotactile neurons, which did not respond to visual stimuli at a far location before training, responded to those far objects after training. Accordingly, objects that were perceived as being in the EPS before tool use, were perceived as being in the PPS after tool use.

As a consequence, using a tool also modifies the representation of the PPS. As evidence, Bourgeois et al. (2014) demonstrated that the area at which objects were considered within reach receded in space after using a tool that provided a functional extension to the arm, but not after using a tool that provided no functional extension to the arm (e.g., a tool with a short handle). The selectivity of these findings provided a compelling demonstration that, for the tool to be effective in shaping the PPS, a functional benefit to the arm is necessary (Witt et al., 2005). To obtain these effects, however, the use of the tool is not always mandatory. For instance, Costantini et al. (2014) showed that passively holding a tool while observing someone else using the same tool led also to an increase in the PPS representation in the observer. Likewise, it is worth noting that remote control situation can also affect the representation of the PPS. For instance, holding a computer mouse that controls visual information on a far computer screen during a multisensory integration task was found to extend the representation of the PPS (Bassolino et al., 2010).

Interestingly, not only the somatotopic representation of the body but also the way mechanical constraints prevent natural movements of body segments may contribute to the representation of the PPS. Immobilizing an upper-limb for 24 h, for instance, produced a significant effect on cortical excitability of the motor regions dedicated to the control of that particular upper-limb (Facchini et al., 2002; Huber et al., 2006; Avanzino et al., 2011). Transient immobilization consequences also reflected in a reduction of movement spatial accuracy (Huber et al., 2006), coordination (Moisello et al., 2008), as well as in motor imagery tasks (Toussaint and Meugnot, 2013; Meugnot et al., 2014; Meugnot and Toussaint, 2015). Toussaint et al. (2018) showed that the plasticity of the sensorimotor system, resulting from the immobilization condition, had a concomitant effect on the PPS representation. They reported shrinkage of the PPS representation following the immobilization period, which was not observed in the non-immobilized condition. Moreover, no effect of arm immobilization was observed in non-spatial perceptual tasks, suggesting herein the absence of specific visual perception or decisional deficits in the limb immobilization condition (Toussaint et al., 2018). Along the same lines, it was found that the PPS representation was narrower in participants wearing wrist weights as if previous reachable objects became suddenly hardly reachable (Lourenco and Longo, 2009). These findings, confirmed in other studies (e.g., Bassolino et al., 2012), validated the crucial role of not only the body schema but also a properly functioning sensorimotor system in the representation of the PPS.

A similar outcome was achieved in a clinical population characterized by hemiplegia, which resulted from lateralized brain stroke (Bartolo et al., 2014). Patients with hemiplegia are indeed specifically impaired in motor imagery task involving their hemiplegic arm, and also when performing a motor imagery task or an actual motor task involving their healthy arm. This pattern of results was however observed in right-brain-damaged patients, not in left-brain-damaged patients, although characterized by the same level of hemiplegia in the contralesional arm. These findings support the hemispheric theory of motor control according to which the right hemisphere predominantly contributes to the specification of the spatiotemporal characteristics of a motor response, whereas the left hemisphere predominantly contributes to the online control of motor execution, especially in the context of complex motor skills (Sainburg et al., 1999; Sainburg and Kalakanis, 2000; Sainburg, 2005; Schaefer et al., 2007, 2009). Supporting this hemispheric framework, Bartolo et al. (2014) showed that patients with right hemisphere brain damage had much more difficulties in representing accurately their PPS, which was characterized by a broad underestimation, even when referred to the healthy arm. These data provided thus compelling evidence that the processes associated with the planning of motor action contribute also to the representation of the PPS. Similar results were obtained in patients showing visuomotor deficits such as optic ataxia, a pathology that occurs following damage to the posterior parietal cortex and which is characterized by a deficit in reaching to visual targets with no isolated signs of perceptual or motor deficits (Bartolo et al., 2018). The latter observations highlighted that the representation of the PPS is a dynamic representation that involves the real-time computation of sensory and motor inputs. Supporting this view, when arm amputees wear their prosthetic arm, their PPS representation stretches farther away (Canzoneri et al., 2013). Overall, these findings revealed that PPS representation is continually updated on the basis of real-time processing of current motor capacities and fast adaptation of the body schema. Transient increase (or reduction) in motor abilities affects the body schema, which in turn affects the representation of the PPS. This reinforces the hypothesis that there is a special link between the PPS representation and the action system. However, besides the body schema and the motor repertoire, the third component of PPS representation must be taken into account, which is related to the affective valence of the objects structuring the environment.

## The Enhanced Perceptual and Cognitive Processing in The Pps

The importance of PPS for interacting with the environment makes it a special spatial area for the processing of manipulable objects. Accordingly, specific brain regions have been identified that specifically respond to the presence of objects in PPS. One particular brain area, thought to selectively encode objects within the range of arm action, is the superior parietal occipital cortex (SPOC). This region, located in the dorsal stream of the visual system (Goodale and Milner, 1992; Milner and Goodale, 1995), shows specific responses to the sight of reachable 3D objects, even when no explicit action is required (Prado et al., 2005; Cavina-Pratesi et al., 2007; Gallivan et al., 2009, 2011). Interestingly, SPOC activation is modulated by gaze distance, which may provide the dorsal stream with accurate information about object localization for action (Culham et al., 2008). To compute real-world distance, the visual system needs indeed real-time information about where the eyes are directed. This information emerges from the combination of retinal signals, eyes-related motor signals (vergence and accommodation), and proprioceptive signals associated with the orientation of the eyes, as well as from information about the location of the target with respect to the gaze (retinal signals and binocular disparity). The modulation of SPOC activity by gaze distance and direction represents thus a key component for computing the object’s location in relation to the PPS, by providing accurate visual orientation and depth information. The SPOC region may thus be a key node within the dorsal stream for the computation of object egocentric location, but also objects affordances, as needed to guide voluntary motor actions. Concerning the specific coding of objects located in the PPS, we may thus expect specific enhancement of their visual perception.

Tackling the issue of perceptual performance in the PPS, several studies have indeed revealed that object processing is improved when located in PPS. For instance, participants make fewer errors and are faster in judging the properties of a visual stimulus when it is located near the body (Reed et al., 2006; Dufour and Touzalin, 2008). Other compelling arguments came from the study by Gori et al. (2011), which evaluated size visual discrimination when presenting spherical objects inside and outside the PPS. These spheric objects were compared to another object presented as a *standard* stimulus and located at the boundary of the PPS. The authors found that visual judgments inside the PPS were more accurate, whereas those outside the PPS were biased towards underestimation. They also showed that the perceptual biases for objects out of reach were significantly reduced when allowing the participants to touch the object without seeing it, after it had been observed, making thus possible a sensorimotor calibration mediated by the haptic signals. They also revealed that observing a confederate grasping the object in the space out of reach also restored accurate size perception. This indicates that haptic information, probably obtained during observation through the mirror system, can compensate for the inaccurate visual information. Other studies revealed also that shape discrimination (Spence et al., 2004; Costantini et al., 2010) or object categorization (Blini et al., 2018) were facilitated when objects were located in the PPS. For instance, Costantini et al. (2010) showed that object-oriented hand movements were facilitated when the imperative visual signal used to trigger the action had a congruent orientation (e.g., using a cup with a handle directed towards the moving arm as a visual stimulus to trigger the action). The perceptual facilitation due to the spatial alignment effect was however observed only for stimuli located in the PPS.

Facilitation of visual processing of objects in the PPS was also found in tasks where no object-oriented responses were requested. In a task requiring to discriminate between 3D visual sphere and cube shapes seen within an immersive virtual reality scene, Blini et al. (2018) showed facilitation of shape discrimination for objects located in the PPS. The perceptual facilitation effect was observed even when controlling for retinal size, binocular cues, and upper/lower visual field, firmly indicating that stimuli in the PPS benefit from enhanced perceptual processing. The increased attention given to the PPS could also lead to bias in visual perception, in particular when focusing on dynamic stimuli. For instance, when dynamic events occur within the PPS, predicting collisions represents a fundamental ability for protecting the body, which develops early in infancy (Eilan et al., 1993; Zago and Lacquaniti, 2005). Studies in humans have shown that collision predictions are based on the processing of perceptual information that physically describes the dynamic event. Among them, object position over time plays a crucial role (Proffitt and Gilden, 1989; Gilden and Proffitt, 2014). In a recent study, Iachini et al. (2017) directly explored whether predicting possible collision depends on the location of objects in space. Collision and non-collision events were devised by manipulating independently the velocity and the path of two balls moving one towards the other, either in or out the PPS. The results revealed a lower discrimination ability when predicting collisions for events occurring in the PPS, together with a significant increase in false alarms. Participants erroneously predicted a collision even though it was not physically possible. Because it is particularly difficult to keep track of moving stimuli following different velocities (Pylyshyn, 2004; Fencsik et al., 2007), one interpretation of these findings was that anticipating even erroneously collision event would provide an adaptive advantage by ensuring that individuals are allowed to prepare for appropriate behavior (Neuhoff, 1998, 2001; Vagnoni et al., 2017). The specific way spatio-temporal information is processed may thus reflect the motor, anticipatory and adaptive function of the PPS that works like an alert system for timely preparing reactions to events that occur near the body (Coello and Iachini, 2020). Although these results provide overall evidence for facilitation of temporal and spatial processing of information in the PPS, it is worth noting that this facilitation was not observed for all objects’ attributes, in particular with respect to color or fine-grained visual objects’ attributes (Gozli and Pratt, 2012; Kelly and Brockmole, 2014). This dissociation may be partly explained by the assumption that the representation of the PPS relies predominantly on the dorsal stream of the visual system, which is involved in the guidance of basic actions, whereas the ventral stream dedicated to the processing of objects’ attributes is more involved in the representation of the EPS (Previc, 1998; Milner and Goodale, 2008).

## The Protective Function of The Pps

It seems therefore apparent that the function of the PPS is not only to specify a functional interface between the body and the environment for goal-directed motor action towards reward-yielding stimuli but also to protect the body from potentially harmful stimuli. As mentioned before, the PPS encoding involves multisensory neurons in the parietal and premotor cortex that respond indifferently to either tactile stimulation resulting from the contact of an object with a body-segment, or when the same object was visually located at the vicinity of a body-segment (Rizzolatti et al., 1981; Graziano and Cooke, 2006). The multisensory neuron activity, which was observed in the mere presence of a nearby visual stimulus, was interpreted as an automatic preparation to react to proximal stimuli, in particular when they can be potentially dangerous for the body. In agreement with this, the electric stimulation of the parietal and premotor cortex triggers defensive motor actions performed by the monkeys’ arm or body (Graziano et al., 2002; Cooke and Graziano, 2003). Thus, it seems that any stimulus located in the PPS receives special attention and activates the sensorimotor system, to prepare the body to act adaptively. Supporting this view, the activity of the human posterior parietal cortex was found to increase when threatening stimuli were presented in the PPS (Lloyd et al., 2006). This suggests that this brain area, in addition to subcortical structures that respond specifically to emotional stimuli (Adolphs, 2002), is sensitive to the effective value of visual stimuli located in the PPS (Lloyd et al., 2006). Consequently, the PPS must be viewed as space not only dedicated to object-directed motor actions, but also to defensive behavior to protect the body from external threat. As a consequence, the presence of a threatening stimulus close to the body alters the representation of the PPS. As evidence, Coello et al. (2012) reported a decrease in PPS representation when dangerous objects were presented close to the boundary of the PPS, although only when the dangerous part of the objects was directed towards the participants (e.g., the blade of a knife). This latter result suggests that the degree of threat associated with an object can influence the representation of the PPS, mainly because of the potential positive/negative consequences of acting towards that object, in relation to the control of approach/avoidance behavior. Accordingly, if a stimulus that can potentially be a threat to the body enters the PPS, defense mechanisms are automatically triggered. Because of this established protective role of the PPS, one may expect the PPS representation to play a key role in the control of social interactions.

## The Representation of The Pps in Social Context

As a consequence of its protective function, the PPS operates indeed as an important spatial buffer in the adjustment of social interactions. Social interactions require minimizing interpersonal distances while avoiding invading other’s PPS (Kennedy et al., 2009). The extent of PPS representation can thus be affected by the presence of conspecifics in the nearby space. In this respect, animal studies revealed that when a monkey share with another monkey a portion of its action space in which food is reachable, a specific reduction of the activity in the parietal and prefrontal cortex is observed in the monkey and this later barely tries to get the food (Fujii et al., 2007, 2009). This disappearance of voluntary actions appears in general in the submissive monkey, not the dominant one. However, regardless of dominance issues, this observation strongly suggests that the PPS representation is shaped by the social context. The same result was reported in human interaction tasks. Coello et al. (2018), for instance, provided compelling findings in a cooperative task involving two facing human confederates. The task consisted for the two confederates to select alternatively a series of targets among a set of 36 gray targets, which could individually provide either a one-point reward (the target turned green when selected) or no point (the target turned red when selected). The probability to get a reward was 50% and the targets were randomly distributed across the surface of a touch-screen table. Surprisingly, in the search for reward-yielding targets, participants acted as if they were splitting the workspace into two equal parts, thus avoiding invading the confederate’s PPS. This finding is in line with the observation that in a social context, people tend to automatically shift their attention away from others in order to subjectively increase the social distance and thus reduce the uncomfortable situation and associated physiological responses induced by the presence of a conspecific (Szpak et al., 2015). However, Coello et al. (2018) showed that despite the reduction of the workspace considered by the confederates, the representation of their PPS extended after having performed a cooperative task (Gigliotti et al., 2021).

Taken together, the above results suggest that PPS representation is sensitive to the social context. When individuals cooperate in a task, their respective PPS initially keeps separated as a consequence of the social context, but progressively merge in the course of the cooperation task to finally achieve a shared action space. Congruent findings were reported by Teneggi et al. (2013) who used a multisensory integration task. Although the PPS representation shrank when the multisensory integration task was performed while a confederate was close to the participant, the PPS representation extended after having performed a cooperative task together. This confirms that cooperating in a task or sharing the same objective yields a merging process, resulting in an overlapping of the self and other’s PPS. This phenomenon seemed however to depend on the information that is available about the other’s characteristics. Using a similar multisensory integration task, Pellencin et al. (2017) observed indeed an increase in the PPS representation when interacting with an assumed moral confederate, but not when interacting with an assumed amoral one. Interestingly, Teramoto (2018) also using the same paradigm showed that participants exhibited shorter detection of a tactile stimulus when an approaching visual stimulus was located in their PPS. In contrast, when performing the task with a partner, the participants exhibited shorter detection times both when the approaching visual stimulus was located either in their own PPS or in their partner’s PPS, i.e., located in the participants’ EPS. Overall, these results indicate that humans can access to different PPS representations and/or body-derived attention mechanisms, depending on the selected perspective taking (Teramoto, 2018). Thus, the presence of other people around us and the evaluation of their potential benefit for us may play an important role in the PPS representation.

## The Functional Role of The Pps in The Control of Interpersonal Distance

Social psychology provided evidence that interpersonal distance is a key component of social interactions. Drawing on the work of ethologists such as Hediger (1950); Hediger (1968) who showed that animals maintain a certain distance from each other in ecological conditions, both within and between species, Hall (1966) developed a theory of social distance in humans now known as proxemics. For Hall (1966); “each animal is surrounded by a series of bubbles or irregularly shaped balloons that serve to maintain proper spacing between individuals (pp.10).” Based on his observations, he established that people maintain a certain distance between them, and the adjustment of this distance seems based on a subtle balance between the need to interact efficiently with conspecifics and a variety of other factors that are driven by approach-avoidance motivations (Argyle and Dean, 1965). Hall (1966) distinguishes between an intimate space, in which we can feel the warmth of another person’s body; a personal space, in which we can get close to relatives and mates; a social space, in which we can interact with non-intimate fellows; and a public space, in which large scale communication is possible but where there is no commitment with other people. Intimate and personal space correspond to distances that match the PPS, whereas social and public space corresponds to distances that match the EPS. The extent of these spaces seem precisely tuned because if the inter-individual distance is too large, it might be not suitable for the kind of social interaction expected, and conversely if the inter-individual distance is too short, it might generate discomfort (Sommer, 1959; Hayduk, 1978; Kennedy et al., 2009; Lloyd, 2009). Appropriate interpersonal distance (IPD hereafter) therefore constitutes the foundation of natural social interaction. Beyond facilitating the processing of sensory information during social interactions (Hall, 1966), preferred IPD seems rooted in sensorimotor representations. Indeed, preferred IPD usually corresponds to a distance that is slightly longer than the length of the arms and depends on people’s height (Hall, 1966; Hartnett et al., 1974; Hayduk, 1983; Pazhoohi et al., 2019). Accordingly, IPD must be intrinsically linked to PPS representation. An increasing body of evidence supports this view (Iachini et al., 2014; Quesque et al., 2017; Vieira et al., 2019). For instance, Iachini et al. (2014) showed that IPD was shorter when interacting with human-like stimuli in comparison to non-human-like stimuli, just as the representation of the PPS when tested in a social context (Teneggi et al., 2013). More importantly, Quesque et al. (2017) showed that modifying PPS representation through tool-use altered preferred IPD in a social situation. In their study, participants performed an IPD judgment task in a virtual environment, with human-like stimuli approaching and passing close to them with different shoulder-to-shoulder distances. They reported two main findings. First, they observed that the minimum shoulder-to-shoulder distance tolerated by the participants was as a function of whether the human-like stimuli crossed their median sagittal axis or not. It was indeed two times larger when the human-like stimulus crossed the participant’s median sagittal axis than when it did not, as if the participants were considering as appropriate the trajectories of the approaching human-like stimulus that did not invade their own PPS. Supporting this interpretation, the authors also observed that the minimum shoulder-to-shoulder distance tolerated increased by 20% after having used a long-handle tool. No effect was observed when the participants manipulated a short-handled tool, providing no functional extension to the arm. Overall, these findings demonstrated that IPD and PPS share common motor resources, and provided support to the hypothesis that the PPS representation contributes to the specification of appropriate IPD. This interpretation was corroborated by brain imaging studies which showed that the frontoparietal areas known to be involved in the PPS representation were also activated in social interaction tasks (Holt et al., 2014; Vieira et al., 2019).

## The Ipd Depends on Attributed Valence to Social Stimuli

Assuming that IPD depends on PPS representation does not however provide a complete picture of the spatial organization of social interactions. In particular, it is well known that IPD adjustment critically depends on people’s characteristics. For instance, IPD varies with people’s age and gender. Females prefer larger IPD than males, as well as older persons prefer larger IPD than younger persons (Iachini et al., 2014, 2016; Ruggiero et al., 2017; Sorokowska et al., 2017). Reciprocally, IPD adjustment is sensitive to the perception of others’ characteristics. Physical attributes such as height, age, or gender contribute indeed to the selection of appropriate IPD (Hartnett et al., 1974; Hayduk, 1983; Uzzell and Horne, 2006; Iachini et al., 2016; Pazhoohi et al., 2019). It was, in particular, reported that IPD reduces when both males and females interact with a female; a young or a small person (Iachini et al., 2014; Hecht et al., 2019). Considering personality traits, it was found that IPD is also sensitive to morality judgments (Iachini et al., 2015a; Pellencin et al., 2017; Fini et al., 2020). Preferred IPD particularly enlarges when a person is presented as immoral, whereas it shrinks when a person is described with high moral values (Iachini et al., 2015a). IPD adjustment is also dependent on social factors such as affiliation because we are more likely to get closer to individuals with whom we identify with (Willis, 1966; Leibman, 1970; Tajfel et al., 1971; Workman, 1987; Fini et al., 2020). As a consequence, IPD decreases with in-group members whereas it increases with out-group members (Hall, 1969; Leibman, 1970; Tajfel et al., 1971; Hendricks and Bootzin, 1976; Fini et al., 2020).

However, one of the crucial aspects of social interactions concerns the evaluation of other internal states (Juckel et al., 2018), especially concerning the emotional component. One may indeed expect IPD adjustment to be sensitive to the perceived emotional state in others, as mainly reflected in their emotional facial expressions (Cartaud et al., 2018). This is essentially because of the adaptive value of emotions (Darwin, 1872; Ekman and Friesen, 1971; Ekman and O’Sullivan, 1988; Waller et al., 2017). Indeed, among the non-verbal social cues supporting social interactions (i.e., gaze contact, facial expression, body posture, and gesture), emotional facial expressions represent a key component of IPD adjustment. Studies on the impact of emotional facial expressions on social interactions have shown that detecting individuals’ intent through their facial expression influences approach-avoidance behavior (Keltner et al., 2003; Fiske et al., 2007; Vilarem et al., 2020). In particular, identifying emotional facial expressions helps to determine whether others have positive intentions (as in the case of intimate social relationships) or may represent a potential threat to us. One may thus expect IPD adjustment to be sensitive to the perceived emotional state in others, as reflected by their facial expressions (Ruggiero et al., 2017; Cartaud et al., 2018, 2020). In agreement with this, it was found that positive facial expressions foster approach behaviors, whereas negative facial expressions lead to avoidance and withdrawal, resulting in a decrease or increase in IPD respectively (Lockard et al., 1977; Ruggiero et al., 2017; Vieira et al., 2017; Cartaud et al., 2018, 2020). The increase in IPD with individuals revealing a negative emotional state may represent avoidance reactions aiming at ensuring a larger margin of self-protection (Dosey and Meisels, 1969; Hayduk, 1983; Siegman and Feldstein, 2014). Thus, considering the same location in space, threatening individuals are perceived closer than non-threatening individuals (Cole et al., 2013).

Interestingly, emotional facial expressions contribute to IPD adjustment in relation to neural and physiological reactions. Perceived emotions mediate psychophysiological as well as automatic behavioral responses (Vuilleumier and Pourtois, 2007). Accordingly, spatial closeness in social context produces changes in the body peripheral activity such as an increase in the electrodermal activity (McBride et al., 1965; Aiello et al., 1977; Wilcox et al., 2006), heart-rate (Wieser et al., 2010), and the level of cortisol (Evans and Wener, 2007), which are even amplified in threatening situations (Cartaud et al., 2018, 2020; Ellena et al., 2020). Kennedy et al. (2009) also showed that too close proximity with a non-intimate person triggers a neural activation of the amygdala, a subcortical brain structure playing a crucial role in emotion regulation. This strong emotional reaction conveyed by the amygdaloid complex is thought to contribute to the control of IPD. Supporting this view, individuals with a complete amygdala lesion showed a severe deficit in regulating IPD, being able to stand comfortably at 34 cm from another person while control participants would not tolerate an IPD lower than 64 cm on average (Kennedy et al., 2009).

It is thus possible to envisage that IPD depends on both the representation of PPS and the reaction to the threat within the PPS. Indeed, because PPS plays like a protective buffer area in social contexts, any threatening stimulus located within the PPS would produce a feeling of discomfort associated with a significant increase in the physiological responses. These physiological responses, or their anticipation, could represent the signal used by the central nervous system to specify appropriate IPD in a social context. This is indeed what Cartaud et al. (2018, 2020) recently investigated by using virtual characters displaying different facial expressions. When a virtual character was presented in the participants’ PPS using immersive virtual reality, an increase in the electrodermal activity was observed which was a function of the facial expression of the virtual character, being higher when showing an angry rather than a neutral facial expression. Electrodermal activity was also higher when the human-like stimulus was located in the PPS rather than in the EPS (Cartaud et al., 2018). Furthermore, and more importantly, they observed that the level of electrodermal activity registered when a virtual character was in the participants’ PPS was predictive of the distance selected as the appropriate IPD to interact with the same virtual character. Moreover, the concurrent increase in electrodermal activity and IPD was particularly observed for the virtual characters showing negative facial expressions. This finding demonstrated that preferred IPD can be predicted from the level of threat perceived on the basis of facial expressions (Ruggiero et al., 2017), which correlates with the emotional response triggered by the same facial expressions when presented in the PPS (Cartaud et al., 2018). This indicates that peripersonal-action space and interpersonal social space are coherently sensitive to the emotional state of conspecifics, which could reflect a common adaptive mechanism shared by theses spaces to subtend interactions with both the physical and social environment, along with the protection of the body from external threats.

## A New Theoretical Model for The Control of Ipd in Social Context

Overall, the present review suggests that preferred IPD represents the appropriate spatial distance between individuals that fosters social interactions while ensuring physiological homeostasis. The studies by Cartaud et al. (2018, 2020) demonstrated that the physiological response triggered by a social stimulus when located in the PPS is predictive of preferred IPD when interacting afterward with the same social stimulus. Indeed, because the PPS represents a protective buffer area in social contexts (Graziano, 2017; Serino, 2019), any threatening information located in the PPS would produce a feeling of discomfort associated with a significant increase in the physiological responses (Graziano and Cooke, 2006; Rossetti et al., 2015; Vieira et al., 2019). This is in line with the view that the PPS must be conceived as an area dedicated to the interaction with the environment whose representation relies specifically on defensive mechanisms (Coello et al., 2012; Graziano, 2017; Serino, 2019). The PPS can thus be seen as a no-go zone for social interactions unless an intimate relationship is built making physical contact tolerated. As a consequence, non-intimate social life occurs essentially beyond the boundary of the PPS, that is in the EPS (Hall, 1966). Therefore, as the PPS must remain inviolate despite the social promiscuity often encountered, its boundary contributes to the construction of IPD in association with the emotional valence of the social stimuli (Quesque et al., 2017; Vieira et al., 2019). If the PPS is violated, this triggers a strong discomfort together with a strong physiological response leading in most cases to defensive behaviors (Kennedy et al., 2009; Cartaud et al., 2018, 2020; Vieira et al., 2019; Ellena et al., 2020). These physiological responses, or their anticipation, could thus represent the signal used by the central nervous system to specify appropriate interindividual social distances. This would thus represent the neural mechanism that allows reinstating a feeling of safety and physiological equilibrium.

**Figure 1:**
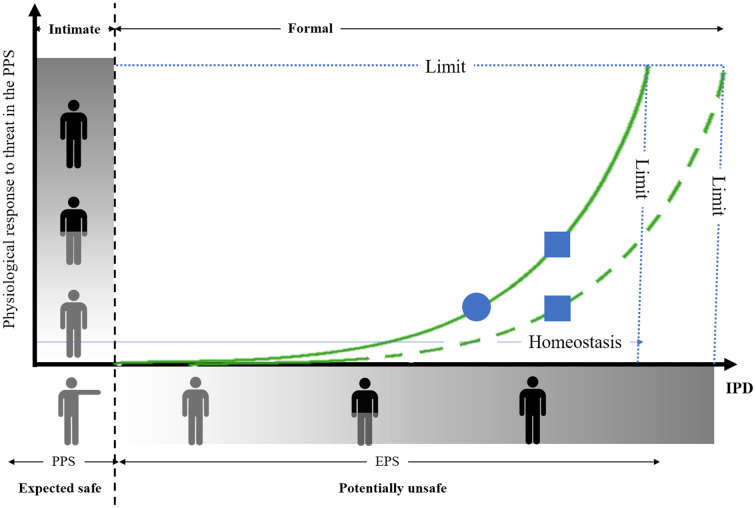
Streamlined representation of the relation between the physiological response to threat in the peripersonal space (PPS) and preferred interpersonal distance (IPD). The origin corresponds to the individual location. The outstretched arm represents the boundary of the PPS (intimate social relationship in the PPS, formal social relationship outside the PPS). The dashed vertical line represents the individual’s PPS. The degree of threat is represented on the axes (no threat: gray character, moderate threat: gray and black character, high threat: black character). The vertical strip represents the change in the physiological response depending on the threat. Preferred IPD necessary for homeostasis is represented on the x-axis (short IPD: gray character, moderate IPD: gray and black character, large IPD: black character). The horizontal stripe represents the change in IPD depending on the threat. Note that there is a limit of increase in the physiological response and IPD. The thin horizontal blue line corresponds to the homeostasis sought by adjusting IPD according to the estimated level of threat. The plain green curve represents the relationship between threat estimates in the PPS and preferred IPD in the general population (not to scale). The dash green curve corresponds to a possible alteration in the relation between the physiological response to threat and IPD in the pathological population. Note that when considering a particular case in the general population (circle), the increase in IPD due to socio-emotional deficit in the pathological population (square) can be the result of an over physiological reaction to threat (upper square), while referring to the same function than that in the general population, or the result of a biased relation between the physiological response to threat and IPD (lower square).

Furthermore, previous studies also highlighted that preferred IPD depends on the emotional valence (i.e., the degree of threat) of social stimuli. At a broader negative valence attributed to others corresponds a larger IPD. Both PPS representation and emotional valence of social stimuli are thus integrated to specify the appropriate social distances. As sketched in Figure 1, we propose that IPD is built on PPS representation (in relation to its intrinsic protective value) and contains an extra margin that adapts depending (in particular) on the perceived valence of social stimuli, which requires estimating the level of threat of conspecifics in relation to the representation of the PPS. In case of close social threat, the physiological responses will alter homeostasis increasing the distance with the social stimulus, so that IPD adjustment finally fits with the function linking threat to distance. The original aspect of this modeling is that the physiological responses to the PPS violation by a threatening social stimulus is predictive of preferred IPD with that particular social stimulus. Preferred IPD would thus correspond to the minimum distance necessary to maintain homeostasis. Accordingly, we propose that IPD is constrained by the relationship linking spatialization to homeostasis, which includes the capacity to represent both the PPS and the emotional valence or threat level of a particular social stimulus.

## Applications to The Clinical Population

The model presented here provides a relevant theoretical framework for interpreting the consequence on IPD of socio-emotional deficits in pathological populations. For instance, several studies reported a correlation between the level of social anxiety and preferred IPD (Dosey and Meisels, 1969; Brady and Walker, 1978; Iachini et al., 2015b; Givon-Benjio and Okon-Singer, 2020). It has indeed been noted a prevalence of enlarged IPD in anxiety dependent psychopathological disorders such as social anxiety (Dosey and Meisels, 1969; Brady and Walker, 1978), claustrophobia (Lourenco et al., 2011), borderline personality disorder (Schienle et al., 2015), autistic spectrum disorders (Perry et al., 2015; Candini et al., 2017), schizophrenia (Horowitz et al., 1964; Schoretsanitis et al., 2016), anorexia (Nandrino et al., 2017), or even cynophobic-based anxiety (Taffou and Viaud-Delmon, 2014). On the contrary, disorders associated with a lack of empathy or with antisocial behaviors such as psychopathy lead to shorter IPD compared to the general population (Rimé et al., 1978; Vieira and Marsh, 2014; Welsch et al., 2018). Interestingly, and in agreement with our model, Kennedy and Adolphs (2014) observed that the significant correlation between (peri)personal space and IPD preferences in a healthy population was altered in adults with diagnosis of autism spectrum disorder. The interpretation of the authors was that alteration of the relationship between (peri)personal space and IPD may relate directly to amygdala dysfunction, a brain region known to contribute to emotional regulation (Kennedy et al., 2009) and presenting anatomical and functional abnormalities in autistic spectrum disorder. Of interest, the non-pathological population characterized by an enlarged self-representation of the PPS was also characterized by a higher rate of social anxiety (Iachini et al., 2015b) and phobia (Lourenco et al., 2011). These findings are thus consistent with a basic motor function of the PPS which subtends a specific role in protecting the body from external hazards (Graziano, 2017), organizing object-directed actions, and regulating our social life (Coello and Iachini, 2016). The model we propose provides a new framework that allows predictions relating to social behavior in the context of socio-emotional pathologies. In particular, compared to a putative performance in the general population (circle on the plain curve, Figure 1) atypical increase in preferred IPD in the pathological population (such as in anorexia or schizophrenia) could result from either a higher physiological response to a similar level of threat (square on the plain curve, Figure 1) or from a biased relation between physiological responses to threat and preferred IPD (square on the dashed curve, Figure 1). Further research would be necessary to disentangle these two possible interpretations of pathological social behavior.

To sum up, the data presented in this position article suggest that the PPS shall be viewed as a dynamic representation of the space around the body subserving primarily the organization of goal-directed behavior towards stimuli endowed with the highest reward value. It must also be viewed as space where potentially harmful stimuli receive specific attention to protecting the body from the hazards ahead. Accordingly, stimuli in the PPS receive particular attention that fosters perceptual and cognitive processes. The PPS represents thus a mediation zone between the body and the environment, protecting the body from external threats and, as such, contributing to the organization of the social life. We also showed that the latter requires the integration of the PPS representation with an estimate of the level of the threat inherent to the social stimuli present in the environment. The model we propose may thus account for deviants in social interaction in pathological populations, in particular when emphasizing specific socio-emotional deficits.

## Author Contributions

All authors listed have made a substantial, direct and intellectual contribution to the work, and approved it for publication.

## Conflict of Interest

The authors declare that the research was conducted in the absence of any commercial or financial relationships that could be construed as a potential conflict of interest.
